# Systematic Review of Health Economic Evaluations of Diagnostic Tests in Brazil: How accurate are the results?

**DOI:** 10.6061/clinics/2017(08)08

**Published:** 2017-08

**Authors:** Maria Regina Fernandes Oliveira, Roseli Leandro, Tassia Cristina Decimoni, Luciana Martins Rozman, Hillegonda Maria Dutilh Novaes, Patrícia Coelho De Soárez

**Affiliations:** I Faculdade de Medicina, Universidade de Brasilia, Campus Universitario Darcy Ribeiro, Brasilia, DF, BR; II Departamento de Medicina Preventiva, Faculdade de Medicina FMUSP, Universidade de Sao Paulo, Sao Paulo, SP, BR; III Hospital de Transplantes Euryclides de Jesus Zerbini, Sao Paulo, SP, BR; IV Instituto de Avaliacao de Tecnologias em Saude (IATS/CNPq), Porto Alegre, RS, BR

**Keywords:** Costs and Cost Analysis, Cost-Benefit Analysis, Health Care Costs, Diagnostic Tests Routine, Brazil

## Abstract

The aim of this study is to identify and characterize the health economic evaluations (HEEs) of diagnostic tests conducted in Brazil, in terms of their adherence to international guidelines for reporting economic studies and specific questions in test accuracy reports. We systematically searched multiple databases, selecting partial and full HEEs of diagnostic tests, published between 1980 and 2013. Two independent reviewers screened articles for relevance and extracted the data. We performed a qualitative narrative synthesis. Forty-three articles were reviewed. The most frequently studied diagnostic tests were laboratory tests (37.2%) and imaging tests (32.6%). Most were non-invasive tests (51.2%) and were performed in the adult population (48.8%). The intended purposes of the technologies evaluated were mostly diagnostic (69.8%), but diagnosis and treatment and screening, diagnosis, and treatment accounted for 25.6% and 4.7%, respectively. Of the reviewed studies, 12.5% described the methods used to estimate the quantities of resources, 33.3% reported the discount rate applied, and 29.2% listed the type of sensitivity analysis performed. Among the 12 cost-effectiveness analyses, only two studies (17%) referred to the application of formal methods to check the quality of the accuracy studies that provided support for the economic model. The existing Brazilian literature on the HEEs of diagnostic tests exhibited reasonably good performance. However, the following points still require improvement: 1) the methods used to estimate resource quantities and unit costs, 2) the discount rate, 3) descriptions of sensitivity analysis methods, 4) reporting of conflicts of interest, 5) evaluations of the quality of the accuracy studies considered in the cost-effectiveness models, and 6) the incorporation of accuracy measures into sensitivity analyses.

## INTRODUCTION

Diagnosing is a crucial process that is the basis of clinical practice and individual care. In the field of collective health, diagnosis also serves as the basis for decisions relating to numerous strategies, such as the implementation of screening, the control of epidemic outbreaks or the organization of secondary prevention actions 1.

Scientific research in the field of diagnosis involves estimating the accuracy and reliability of measurements. The costs of diagnostic tests should also be estimated, especially when new technologies that impose higher costs on health systems are involved.

Coverage and reimbursement for diagnostic tests face much higher entry barriers. The lack of evidence about health and economic outcomes has been cited as a common reason for unfavourable coverage decisions 2. Health technology assessment agencies worldwide are increasingly using information from health economic evaluations (HEEs), and they have intensified their scrutiny of diagnostic tests 3. In Brazil, HEEs constitute a relatively new area of scientific research 4.

In 2011, the National Committee for Health Technology Incorporation of Sistema Único de Saúde (Brazilian Public Health System [SUS]), CONITEC, began requiring HEEs to either help inform policy recommendations for the adoption of new technologies or review the policy recommendations made by SUS 5. From 2012 to 2015, CONITEC approved the incorporation of 15 new diagnostic procedures, including Xpert *Mycobacterium tuberculosis* (MTB)/rifampicin (RIF) for tuberculosis diagnosis and positron emission tomography (PET)/computed tomography (CT) for the diagnosis and staging of colorectal and lung cancer and the evaluation of treatment response in Hodgkin’s and non-Hodgkin's lymphoma. Diagnostic procedures represented 17% (15 of 88) of the new technologies incorporated during this period.

Some Brazilian HEEs have analysed the efficiency of diagnostic technologies, similar to those performed in other countries 6-9; however, the characteristics of the body of HEEs related to diagnostic tests in Brazil remain unknown.

This systematic review focuses on the production of HEEs in the field of diagnosis and examines how economic models are implemented in the diagnostic area, thereby identifying methodological gaps that can be addressed in future studies. It is of particular interest to examine if and how test accuracy has been incorporated into economic evaluation models.

Therefore, the objective of this study was to identify and characterize the HEEs of diagnostic tests conducted in Brazil in terms of their adherence to international guidelines for reporting economic studies and specific questions in test accuracy reports over an extended period.

## MATERIALS AND METHODS

This systematic review forms part of a larger research project that systematically reviewed all HEEs related to Brazil that were published between 1980 and 2013 10. This study is in accordance with the guidelines for the systematic review of HEEs published by the UK National Health Service (NHS) Centre for Reviews and Dissemination 11.

### Study identification

We searched multiple databases, including Medline (via PubMed), *Excerpta Medica*, the Latin American and Caribbean Health Sciences Literature database, the Scientific Electronic Library Online, the database of the Centre for Reviews and Dissemination, the NHS Economic Evaluation Database, the NHS Health Technology Assessment database, Bireme and the Health Economics database of the Brazilian Virtual Library of Health. We searched the following citation indexes: Scopus, Web of Science, and the Brazilian Network for the Evaluation of Health Technologies. We also performed manual searches based on the reference lists of included articles and all issues of the Brazilian Journal of Health Economics (BJHE), which was a non-indexed journal in the databases mentioned above in 2013. The search strategy combined subject headings (Medical Subject Headings [MeSH] and Embase subject headings [EMTREE]) and free text terms (“Health Economics” OR “Economics, Hospital” OR “Economics, Medical” OR “Economics, Nursing” OR “Economics, Pharmaceutical” OR “Economics” OR “costs and cost analysis” OR “Cost” OR “Cost savings” OR “Cost of illness” OR “Analyses, Cost-Benefit” OR “Analysis, Cost-Benefit” OR “Cost-Benefit Analyses” OR “Cost Benefit Analysis” OR “Analyses, Cost Benefit” OR “Analysis, Cost Benefit” OR “Cost Benefit Analyses” OR “Cost Effectiveness ” OR “Effectiveness, Cost ” OR “cost effectiveness analysis ” OR “cost-Benefit Date” OR “cost Benefit Date” OR “Date, Cost-Benefit” OR “cost Benefit” OR “Benefits and Costs” OR “Costs and Benefits”) for the “economic/cost” concept with subject headings (MeSH and EMTREE) and free text terms (“Brazil” OR “Brazilian” OR “Brazi*”) for the “Brazil” concept. All searches were limited to 1980-2013. The Medline full electronic search strategy is described in Appendix 1.

### Study selection

Articles were included if they were partial or full economic evaluations, according to the classification devised by Drummond et al. 12, if they addressed a diagnostic test, if they were conducted in a Brazilian setting, and if at least one of the authors was affiliated with an institution in Brazil.

Studies were considered partial HEEs if they examined only costs (cost description), described the costs of a particular disease to society (cost of illness), described the costs and outcomes of a single service or programme (cost-outcome description), described the financial consequences of technology adoption (budget impact analysis [BIA]) or compared only the costs of two or more interventions (cost analysis). Studies were considered full HEEs if they compared the costs and consequences of two or more health care intervention alternatives, including cost-consequences analysis (CCA), cost-minimization analysis (CMA), cost-effectiveness analysis (CEA), cost-utility analysis (CUA) and cost-benefit analysis (CBA).

### Data extraction

Two reviewers (TCD and RL), working independently, selected studies and extracted data using a template developed specifically for this study. The data extracted from each study included the following: year and journal of publication; type of economic evaluation; category of diagnostic test (laboratory tests, imaging, rapid tests or microorganism culture); purpose of the technology assessed (diagnosis, screening, or treatment); the type of affiliation of the first author; the geographical location of the first author; conflicts of interest, as defined by Valachis et al. 13; estimates of cost-effectiveness; and the conclusion of the study (favourable, unfavourable, or neutral). For full HEEs, we also recorded whether the studies contained information on diagnostic test accuracy to answer the following questions: a) Was the accuracy of each test defined and incorporated into the model? b) Were the test accuracy parameters subjected to any sensitivity analysis? c) Was the quality of the evidence from the primary research used to estimate the model parameters of the accuracy test subjected to a quality assessment? Disagreements regarding the extracted data were resolved by consensus or through consultation with a third reviewer (PCS).

For studies in which the cost year was not specified, we assumed that the cost year was the same as the year of publication, a strategy that has been adopted in previous reviews of the literature 14. The monetary values of the results were compared to the year’s Brazilian Produto Interno Bruto (gross domestic product [PIB]) per capita, which is available from the Brazilian Institute for Geography and Statistics 15.

### Quality assessment

The methodology for the quality reporting of individual studies was assessed using some items of the Consolidated Health Economic Evaluation Reporting Standards (CHEERS) 16.

### Data synthesis

A qualitative narrative synthesis was conducted, and the study characteristics are summarized in the figures and summary tables.

Data analysis was performed with descriptive statistics, such as absolute frequencies (raw counts) for each category of the discrete variable and relative frequencies (proportions or percentages of the total number of observations).

## RESULTS

**Figure 1:**
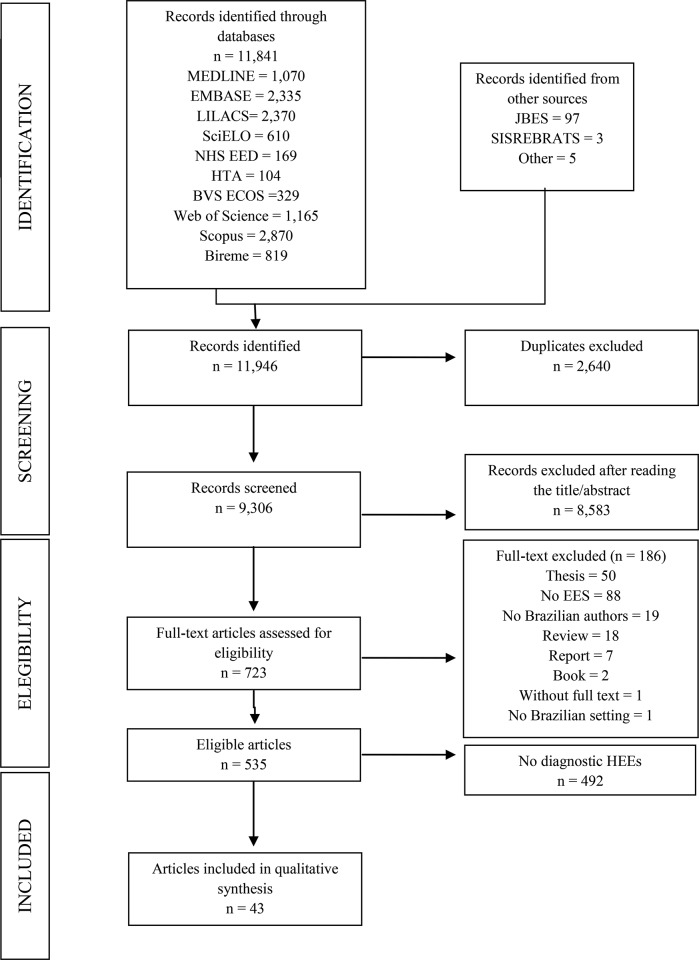
Flow diagram of the process used to select HEEs related to diagnostic tests in Brazil, 1983-2013. *HEEs: health economic evaluations.

**Figure 2:**
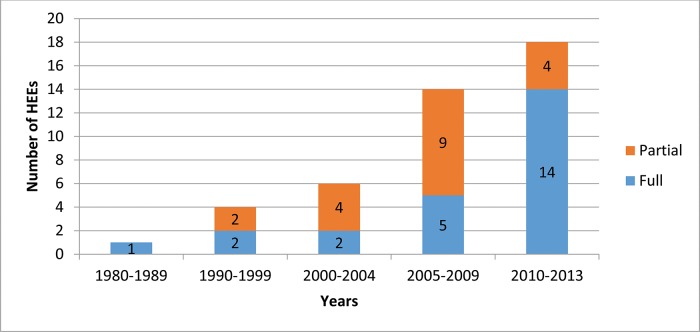
Growth of HEEs related to diagnostic tests (n=43) in Brazil, 1980-2013.

We identified 535 HEEs related to Brazil that were published in 1980-2013 (Figure 1). Of those 535 studies, 43 (8%) addressed diagnostic tests 17-59. The number of studies and the proportion of full HEEs relative to the proportion of partial HEEs increased during the period under study (Figure 2). Of the 43 HEEs related to diagnostic tests, the majority (74.4%, 32/43) were published in the last ten years (i.e., between 2005 and 2013), and 19 (44.2%) were full HEEs.

Table 1 shows an overview of the main characteristics of the diagnostic tests addressed in the 43 selected studies. The technologies were applied to multiple clinical areas, including oncology, cardiology, endocrinology and infectious diseases. The most commonly evaluated tests were laboratory tests (37.2%, 16/43) and imaging tests (32.6%, 14/43). Most were non-invasive tests (51.2%, 22/43) and were utilized in the adult population (46.5%, 20/44). In terms of the intended purposes of the technologies evaluated, most (69.8%, 30/43) were diagnostic; diagnostic and treatment accounted for 25.6% (11/43), and screening, diagnostic and treatment accounted for 4.7% (2/43).

The most common study design was CEA (27.9%, 12/43), and the least common were CUA (2.3%, 1/43) and CMA (2.3%, 1/43). Among the partial HEEs, the most common study design was cost-analysis (25.6%, 11/43), followed by cost-description (18.6%, 8/43).

The majority (55.8%, 24/43) of the HEEs were published in national journals. Analysing the geographic distribution of the affiliations of the first authors revealed a concentration of HEEs from south-eastern Brazil, which accounted for 74.4% (32/43) of the studies identified. The first authors had the following types of affiliations: academia (62.8%, 27/43), healthcare facilities (23.2%, 10/43), research institutes (4.7%, 2/43), consultancies (4.7%, 2/43), and public administration (4.7%, 2/43).

Twenty-four (55.8%) of the 43 studies published statements of financial support. Most of them received financial support from funding agencies (41.6%, 10/24) and governments (25.0%, 6/24). The remaining 19 articles (44.2%) contained no information regarding financial support.

The authors declared conflicts of interest in 16 studies. However, when we evaluated the studies using the criteria proposed by Valachis et al. 13, we identified conflicts of interest in two additional studies. These conflicts of interest could include receiving remuneration in payment or in kind (e.g., stocks or shares) from the manufacturer as a result of any of the following: research support or employment contracts (salary, equipment, supply, reimbursement for participation in symposia and other expenses) or consulting services. In the article by David-Neto et al. 22, we identified manufacturer financing, which was not disclosed as a conflict of interest in the original article. Additionally, in the article by Bacchi et al. 43, we noted that some of the authors were employed by consulting firms, and this was not disclosed as a potential conflict of interest in the original article.

Table 2 presents the main methodological characteristics of the 24 full HEEs, and Table 3 lists their compliance with recommendations for good reporting.

Ten (41.7%) of the 24 full HEEs reported their main outcome to be measurements related directly to the diagnosis strategy, for example, “case diagnosed”. The others evaluated consequent diagnostic outcomes, such as case averted, surgery averted, complication averted, life years saved, and disability-adjusted life years (DALYs) avoided (Table 3). In one of the HEEs 26, the incremental cost-effectiveness ratio (ICER) reported was above the threshold of three times the per capita gross domestic product (GDP) per DALY gained, and the conclusion of that study regarding the technology was unfavourable (Table 3). Three studies 39, 54, 59 presented as their conclusion the measurement of the mean cost-effectiveness ratio (CER), and two studies 34, 52 only mentioned that the strategy was dominant without presenting ICER calculations.

Most studies clearly stated the research question (100%), target population (95.8%, 23/24), competing alternatives (100%), source of effectiveness estimates (95.8%), source of cost estimates (87.5%, 21/24), and ICER (83.3%, 15/18). However, only 12.5% (3/24) described the methods used to estimate the quantities of resources in a correct and transparent manner and reported them separately from their prices (unit costs). Only 29.2% (7/24) noted the type of sensitivity analysis, and 33.3% (6/18) described the discount rate applied.

In relation to the measurements of diagnostic effectiveness, among the 12 CEAs, nine studies (75%) listed the measurements of accuracy (sensitivity and specificity) as epidemiological parameters of the model, and in five of them (55%), the variations in the accuracy measurements were incorporated into the sensitivity analysis. The only CUA, which used DALY as an outcome, applied the estimates of sensitivity and specificity as epidemiological parameters in the decision model and the variations in these measures in the sensitivity analysis. Two studies (17%) referred to the application of methods defined in the literature to check the quality of the accuracy studies that underpinned the economic model 44, 53.

## DISCUSSION

To the best of the authors’ knowledge, this is the first systematic review of Brazilian HEEs in the area of diagnostic technologies. These HEEs were applied to diverse fields, including oncology, cardiology, infectious diseases and endocrine and metabolic diseases. The studies presented results that are relevant for decisions taken in clinical and public health situations in terms of diagnosis (69.8%), and some analyses were relatively broad, integrating screening and/or treatment strategies with diagnostic methods.

Regarding the quality of reporting, the Brazilian HEEs devoted to diagnostic testing performed reasonably well. Almost all studies satisfactorily stated the research question (100%), target population (95.8%, 23/24), competing alternatives (100%), source of effectiveness estimates (95.8%), source of cost estimates (87.5%, 21/24), and ICER (83.3%, 15/18). Strong compliance with HEE guidelines was also reported in a recent study that systematically reviewed all CUAs addressing diagnostic laboratory testing in the Tufts Medical Center Cost-Effectiveness Analysis Registry 3. Nevertheless, some issues still warrant further consideration. First, only 55.8% (24/43) of the studies declared the funding source, and second, only 37.2% (16/43) of the studies declared the potential for any conflict of interest. Missing details on these two important topics may affect the credibility and transparency of the results produced by HEE studies.

Three points were identified as requiring special attention and recommendations for improvement: 1) methods for estimating resource quantities and unit costs, 2) sensitivity analysis, and 3) discount rates. A previous review on the methodological issues of HEEs reached a similar conclusion in the 1990s, observing that many studies failed to report important issues, such as the study’s perspective and sensitivity analyses 60.

Brazil published its official health technology assessment guidelines in 2009. Additionally, in 2011, the use of HEEs became compulsory for the incorporation of new technologies in the Brazilian public healthcare system. However, a clearly stated “reference case” that would increase the consistency of HEE methodology remains lacking. Despite the substantial number of HEEs produced 61, the local capacity to develop and use high-quality HEEs still needs to increase. The lack of technical expertise and the shortage of trained health economists were also reported in other middle-income countries (MICs) 62.

Challenges facing those conducting and using economic evaluations in many MICs include the scarcity, quality and accessibility of data; insufficient health economic research capacity; and limited funding. To conduct high-quality HEEs, far more investment in the training of researchers is needed, particularly for Master’s degree programmes and topics such as study design, data collection and analysis. On-the-job training and mentoring of researchers are also very important and should be fully funded 63.

Diagnostic validity is the definitive stage of technology evaluations in terms of the probability of true positives (sensitivity) and true negatives (specificity) relative to a gold-standard technique. Indeed, measurements of validity are the principal data that determine the effectiveness of economic models 64.

Most of the CEAs (75%) reviewed clearly expressed the measurements of sensitivity and specificity used in the economic model, even when the final outcomes of the economic analysis were not accuracy as such (i.e., in terms of accurate or appropriate diagnoses) but were, instead, measurements arising from accuracy, such as deaths prevented.

Published accuracy studies should be rigorously evaluated for their quality before they can be sued to support the development of economic models. Two published sets of guidelines underpin this quality analysis: Quadas 2 and Stard 65, 66; the former is especially directed towards evaluations in systematic reviews, whereas the latter is directed towards reporting in editorial terms. In this review, only two of the 12 HEEs (17%) cited the quality evaluation of the accuracy studies from which the parameters of sensitivity and specificity were extracted. This is an important omission in these publications and should be part of the reports because these are basic and essential measurements in cost-effectiveness results and studies that discuss the costs and consequences of diagnostic technologies. Indeed, accuracy measurements are the main parameters that direct the analytical model, and therefore, methodological weakness in the estimation of measurements will directly impact the conclusions of the economic evaluation 9. Furthermore, evaluations of the quality of the studies underpinning economic models should be explicit and debated in a transparent manner 67. These parameters must be subject to sensitivity analysis because slight variations can strongly affect the economic conclusions 44.

A review of HEEs of the diagnosis of chronic renal disease suggested several topics that should be addressed when conducting economic studies on diagnosis: specification and clear definition of the measurements of the sensitivity and specificity of the tests and consideration of the consequences of including incorrect results in the economic model 6.

In the research protocol “economic decision models for genomic testing strategies”, Peters et al. (2015) commented that it is rare for economic studies to provide suitable information about the methods used to evaluate how the evidence was identified 7. A similar situation was observed in this review, since only two of the 12 articles on cost-effectiveness that were reviewed actually described the evaluation process used to determine the quality of the primary studies 44, 53.

Otero et al. reviewed HEEs on diagnoses based on images and determined that the accuracy measurements were explained in most of the studies evaluated; however, in 23% of the studies, these measurements were not submitted to a sensitivity analysis. In the Brazilian review, five out of 12 cost-effectiveness studies reviewed and the only CUA varied in the accuracy measurements used in the sensitivity analysis. It should be stressed that this finding should be 100% 8.

This review demonstrates that Brazilian HEEs related to diagnostic tests are reasonably adherent to key recommended general methods for HEEs. However, quality evaluations of the accuracy studies from which the parameters of sensitivity and specificity are extracted is far from meeting current international standards. If these studies are to serve as an important source of information for local decision makers, important issues need to be addressed. First, they should improve the reporting of the methods used to estimate resource quantities and unit costs, sensitivity analysis, discount rate, and conflicts of interest. Second, the evaluation process used to determine the quality of the accuracy studies should be explicit and transparent, and the sensitivity analysis of sensitivity and specificity parameters should be clearly described. Finally, the accuracy measurements are the main parameters that impact decision analysis models’ results and the conclusions of HEEs. Thus, they must be both valid and reliable to support decisions about the incorporation of new diagnostic technologies in Brazil.

### Key Points for Decision Makers

- The Brazilian HEEs in the area of diagnostic technologies require improvement in the quality of reporting of 1) methods used to estimate resource quantities and unit costs, 2) discount rates, 3) descriptions of sensitivity analysis methods, and 4) the reporting of conflicts of interest.
- Decision-making regarding the inclusion of diagnostic technologies should take into account the quality of the accuracy studies that underpin the economic model and the sensitivity analyses of sensitivity and specificity parameters.
- The sensitivity and specificity parameters must be both valid and reliable if they are used to support decisions about the incorporation of new diagnostic technologies in Brazil.

## AUTHOR CONTRIBUTIONS

Decimoni TC and De Soárez PC designed the research. Leandro R, Decimoni TC and Rozman LM performed the research. Oliveira MR, Leandro R, Novaes HM and De Soárez PC analysed the data. Oliveira MR, Novaes HM and De Soárez PC wrote the manuscript.

## APPENDIX 1. MEDLINE (PUBMED) SEARCH STRATEGY

**Table:** 

#1	"Costs and Cost Analysis"[Mesh]
#2	"Economics, Hospital"[Mesh]
#3	"Economics, Medical"[Mesh]
#4	"Economics, Nursing"[Mesh]
#5	"Economics, Pharmaceutical"[Mesh]
#6	#1 OR #2 OR #3 OR #4 OR #5
#7	pharmacoeconomic*[Title/Abstract]
#8	cost minimization[Title/Abstract]
#9	cost effectiveness[Title/Abstract]
#10	cost benefit[Title/Abstract]
#11	cost utility[Title/Abstract]
#12	cost of illness[Title/Abstract]
#13	cost consequence[Title/Abstract]
#14	health economics[Title/Abstract]
#15	#7 OR #8 OR #9 OR #10 OR #11 OR #12 OR #13 OR #14
#16	#6 OR #15
#17	letter[PublicationType]
#18	editorial[PublicationType]
#19	historical article[Publication Type]
#20	#17 OR #18 OR #19
#21	#16 NOT #20
#22	Brazil[MeSHTerms]
#23	Brazil
#24	brasil[Affiliation]
#25	brazil*
#26	brasil*
#27	brasil[Title/Abstract]
#28	brazil[Title/Abstract]
#29	#22 OR #23 OR #24 OR #25 OR #26 OR #27 OR #28
#30	#21 AND #29

## Figures and Tables

**Table 1:** General characteristics of HEEs related to diagnostic tests conducted in Brazil, 1980-2013 (n = 43).

Characteristics	N (%)
Type of diagnostic tests	
Other laboratory tests*	16 (37.2)
Imaging	14 (32.6)
Set of techniques	5 (11.6)
Rapid tests	3 (7.0)
Algorithms	2 (4.7)
Other (program, physical exam)	2 (4.7)
Cultures of microorganisms	1 (2.3)
Type of diagnostic procedure	
Non-invasive	22 (51.2)
Invasive**	20 (46.5)
Does not apply	1 (2.3)
Technology purpose	
Diagnosis	30 (69.8)
Diagnosis and treatment	11 (25.6)
Screening, diagnosis and treatment	2 (4.7)
Target population	
Adults	21 (48.8)
Not declared	17 (39.5)
Children	2 (4.7)
Adults and children	2 (4.7)
> 12 years	1 (2.3)
**Type of study**	
**Full HEEs**	24 (55.8)
CEA***	12 (27.9)
CCA	6 (14.0)
CBA****	4 (9.3)
CUA	1 (2.3)
CMA	1 (2.3)
**Partial HEEs**	19 (44.2)
Cost analysis	11 (25.6)
Cost description	8 (18.6)
**Journal**	
National	24 (55.8)
International	19 (44.2)
**Region of the country of the first author’s affiliation**	
South-east	32 (74.4)
South	6 (14.0)
North-east	2 (4.7)
Centro-Oeste	2 (4.7)
North	1 (2.3)
**Type of institution of the first author’s affiliation**	
Academia	27 (62.8)
Health care facility*****	10 (23.2)
Research institute	2 (4.7)
Consultancy	2 (4.7)
Public administration ******	2 (4.7)
**Source of funding**	
Declared	24 (55.8)
Research funding agency	10 (41.6)
Government	6 (25.0)
Declared no financing	4 (16.6)
Industry	2 (8.3)
International organization	2 (8.3)
**Conflicts of interest**	
Reported	16 (37.2)
Declared no conflicts of interest	16 (100)
Declared conflicts of interest	0 (0.0)
Present, according to Valachis [13]	2 (12.5)
**Study conclusion** *******	
Favourable	17 (70.8)
Neutral	5 (20.8)
Unfavourable	2 (8.3)

* Other laboratory tests: blood test, biopsy, and cytology.

HEEs: health economic evaluations

** Invasive: Those tests that disrupt barriers, such as skin or mucous membranes.

*** One study performed a CEA and a BIA.

**** One study performed a CBA and a CMA.

***** Health care facilities: public or private hospitals, blood centres, and laboratories.

****** Public administration: Ministry of Health, State Health Secretary, and Municipal Health Secretary.

******* This information was extracted only for the full HEEs (n = 24)

**Table 2:** Methodological characteristics of the included full HEEs (n = 24)

Study	Diagnostic test	Comparators	Price year cost	Outcome measures	Study type	Time horizon	Discounting	ICER	ICER classification	Analysis conclusion
Cerci JJ et al. 2012	Metabolic staging with PET (FDG-PET)	Conventional staging*	2010	Surgery avoided Susceptible individuals detected	CCA	NA	NA	NA	NA	Favourable
Koenig A et al. 2010	Protocol for the early detection of sepsis	No protocol	2006	Years of productive life lost	CCA	NA	NA	NA	NA	Favourable
Andrade MV et al. 2011	Electrocardiogram by telecardiology	Electrocardiogram by referral to another municipality	2008	Costs avoided for transport, food, tests and medical appointments	CBA	NI	NI	BCR positive	NA	Favourable
Araújo DV et al. 2008	B-type natriuretic peptide	Clinical judgment	NI	Hospitalization avoided Echocardiogram avoided	CEA	60 days	NI	Dominant	< 1 GDP per capita	Favourable
Barreto AMEC et al. 2008	Algorithms A and B for the diagnosis of hepatitis C virus **	Algorithm C (Conventional algorithm)***	NI	Concordance of results/Diagnostic performance	CCA	NA	NA	NA	NA	Neutral
Bocchi EA et al. 1997	Gallium-67 cardiac scintigraphy	Endomyocardial biopsies	NI	One-year survival, number of ndomyocardial biopsies/patient, treated rejection episodes, tricuspid regurgitation	CCA	NA	NA	NA	NA	Neutral
Camelo Jr JS et al. 2009	Neonatal screening for galactosaemia	No screening	NI	Medical care avoided Improved quality of life	CBA	NI	NI	BCR:1,04	NA	Favourable
Cerci JJ et al. 2011	FDG-PET	Conventional clinical staging methods, including CT, bone marrow biopsy (BMB), and laboratory tests	2009	Modified treatment case	CEA	NI	NI	$16,215	1 to 3 GDP per capita	Favourable
Cerci JJ et al. 2010	FDG-PET + CT + Biopsy	CT + Biopsy	2008	True case detected	CEA BIA	NI	NI	Dominant (-$3,268)	< 1 GDP per capita	Favourable
Bertoldi EG et al. 2012	Annual echocardiogram for all patients Echocardiogram for patients with abnormal levels of B-type natriuretic peptide	No screening No screening	NI	Diagnosis of cardiotoxicity	CEA	NI	NI	Private sector perspective US$19,925.64 to US$30,951.53 Public Sector perspective US$7,668.00 to US$20,232.87	1 to 3 GDP per capita < 1 GDP per capita	Neutral
Oliveira MRF et al. 2010	Optimal ® RDT for malaria	Thick smear microscopy	2006	Cases properly diagnosed	CEA	1 year	Yes	US$549.9	< 1 GDP per capita	Unfavourable
Oliveira MRF et al. 2012	RDTs for malaria	Thick smear microscopy	2010	Cases diagnosed	CEA	1 year	NI	US$44.77	< 1 GDP per capita	Neutral
Dowdy DW et al. 2008	Sputum smear microscopy	Sputum smear microscopy + solid media or liquid media with MGIT	2006	DALY avoided Case avoided	CUA	Lifetime	Yes	US$962	< 1 PIB per capita	Favourable
Oliveira MHP et al. 1983	Self-clean before urine collection	Aseptic technique made by the nursing professional	1982	Resultado microbiológico	CMA	NI	NI	NI	NA	Favourable
Laranjeira FO et al. 2012	Portable monitors of glycated haemoglobin A1c	Conventional laboratory test	2011	Complications avoided	CEA BIA	Lifetime	Yes	Dominant	< 1 GDP per capita	Favourable
Nomelini Rs et al. 2012	PCR for detecting HPV 16/18	Single probe-based PCR	NI	HPV and cervical lesions detected	CCA	NA	NA	NA	NA	Favourable
Pang LW et al. 2001	New malaria management program (community-based diagnosis and treatment)	Old malaria management program (central laboratory visits)	NI	NI	CMA CBA	1 year	Yes	BCR=9:1 Net savings: $60,900	NA	Favourable
Scherer LC et al. 2009	AFB smear used with PCR dot blot	AFB smear used with culture	NI	Case correctly diagnosed	CEA	NI	NI	US$13,749	1 to 3 GDP per capita	Neutral
Silva LK 2003	Bone densitometry, + alendronate sodium or Bone densitometry, + hormone therapy or Hormone therapy or Calcium replacement + vitamin D	Traditional care	NI	Femur fracture avoided	CEA	NI	NI	R$12.408	> 3 GDP per capita	Unfavourable
Vanni T et al. 2011	Strategy A****	Strategies B, C, D and E*****	2008	Life years saved	CEA	Lifetime	Yes	US$10,303.54	1 to 3 GDP per capita	Favourable
Ward LS et al. 1993	Fine needle aspiration cytology	Scintigraphy	NI	Surgery avoided	CBA	NI	NI	Public sector perspectiveBCR= 10.3Private sector perspectiveBCR=8.5	NA	Favourable
Guerra RL et al. 2013	Strategy E1******	Strategies E2 and E3*******	2012	Correct diagnosis	CEA	NI	NI	US$56.69	NI	Favourable
Steffen RE et al. 2013	QFT-GIT Or Tuberculin skin test followed by QFT-GIT	Tuberculin skin test	2010	Case avoided	CEA	2 years	Yes	US$16,021	1 to 3 GDP per capita	Favourable
Rosa RCM et al. 2012	Screening with abdominal ultrasound	No screening	NI	Case identified	CCA	NA	NA	NA	NA	Favourable

CEA: Cost-effectiveness analysis, CCA: Cost-consequence analysis, CBA: Cost-benefit analysis, CUA: Cost-utility analysis, CMA: Cost-minimization analysis, BIA: Budget impact analysis, NI: not informed, NA: not applicable, DALY: disability adjusted life year, BCR: Benefit Cost Ratio, FDG-PET: fluorine-18 (^18^F)-fluoro-2-deoxy-D-glucose–positron emission tomography, CT: computed tomography, RDT: rapid immunochromatographic diagnostic tests, MGIT: Mycobacteria Growth Indicator Tube, PCR: polymerase chain reaction, AFB: Acid fast bacilli smear microscopy by Ziehl-Neelsen staining, QFT-GIT: Quantiferon® - TB Gold-in-Tube, GDP (Gross domestic product) per capita of Brazil, in the price year cost reported by the study. When the study did not report the price year cost, it was assumed to be the year of the publication of the study.

* Conventional staging includes physical examination, laboratory tests (lactate dehydrogenase [LDH], alkaline phosphatase, liver enzymes, bilirubin, renal function, calcium) and CT (chest, abdomen and pelvis).

** Algorithm A: based on a specific level of s/co ratio to determine the need for reflex supplemental testing. The s/co ratio chosen for cut-off (cut-off ratio) corresponded to the ratio that had the highest ≥95% concordance with positive results in the immunoblot (IB) test. Algorithm B: reflex testing by the supplemental nucleic acid amplification test (NAT) was required for samples that were positive or inconclusive in the screening test. A sample was considered as a true positive when both enzyme-linked immunosorbent assay (ELISA) and PCR were positive. When PCR was negative, IB was conducted.

*** Algorithm C: supplemental serologic reflex testing such as IB to confirm screening-test-positive results.

**** Strategy A: Repeat cytology every 6 months. Return to routine screening (every 3 years) only after two consecutive negative cytology results. In the case of a second abnormal smear, patients were referred to colposcopy.

***** Strategy B: colposcopy. Strategy C: HPV testing. In the case of a positive result, they were referred to colposcopy; otherwise, they had to repeat cytology. Strategy D: If the women were 30 years old or older, they were referred to colposcopy (as in Strategy B); otherwise, they had to repeat cytology (as in Strategy A). Strategy E: If the women were 30 years old or older, they were referred to HPV testing (as in Strategy C); otherwise, they had to repeat cytology (as in Strategy A).

****** Strategy E1: chest X-ray followed by smear microscopy for patients with chest-X-ray suggestive of tuberculosis;

******* Strategy E2: smear microscopy followed by chest X-ray for patients with one smear-positive or two smear-negative results; Strategy E3: microscopy and chest X-ray at the same visit.

‡ The summary measure is a CER.

**Table 3:** Methodological quality reporting of HEEs related to diagnostic tests conducted in Brazil, 1980-2013 (n = 24).

Items	N (%)
**Study question**	24 (100.0)
**Target population**	23 (95.8)
**Study perspective**	13 (54.2)
Public Health System	7 (53.8)
Health System (public and private)	3 (23.1)
Public Health System and Society	1 (7.7)
Private Health System	1 (7.7)
Society	1 (7.7)
**Comparators**	24 (100.0)
Time horizon (n=18)*	8 (44.4)
Lifetime	3 (37.5)
1 year	3 (37.5)
2 years	1 (12.5)
60 days	1 (12.5)
Discount rate (n=18)*	6 (33.3)
Applied to costs and benefits	3 (50.0)
Declared that did not apply	2 (33.3)
Applied to costs	1 (16.7)
**Sources for effectiveness estimates**	23 (95.8)
**Sources for resource use estimates**	16 (66.7)
**Sources for cost estimates**	21 (87.5)
**Methods for the estimation of quantities and unit costs**	3 (12.5)
Decision analysis model (n=18)*	12 (66.7)
Decision Tree	8 (66.7)
Markov	3 (25.0)
Decision analytical model	1 (8.3)
Not reported	6 (33.3)
**Sensitivity analysis**	7 (29.2)
**Conducted**	12 (50.0)
Univariate	3 (25.0)
Univariate and Multivariate	2 (16.7)
Univariate and Probabilistic	1 (8.3)
Univariate, Multivariate and Probabilistic	1 (8.3)
Not reported	5 (41.7)
ICER presentation (n=18)*	15 (83.3)

* This item does not apply to the six cost-consequence studies.

ICER: incremental cost-effectiveness ratio.
